# Immune Tolerance Regulation Is Critical to Immune Homeostasis

**DOI:** 10.1155/jimr/5006201

**Published:** 2025-01-07

**Authors:** Lei Han, Tianxiang Wu, Qin Zhang, Anning Qi, Xiaohui Zhou

**Affiliations:** ^1^Department of Pharmacy, Jiangsu Health Vocational College, Nanjing 211800, Jiangsu, China; ^2^School of Basic Medicine and Clinical Pharmacy, China Pharmaceutical University, Nanjing 210009, Jiangsu, China; ^3^Medical Laboratory, Liuhe People's Hospital of Jiangsu Province, Nanjing, Jiangsu 211500, China

**Keywords:** AICD, clonal deletion, immune tolerance, TCR, Treg

## Abstract

The body's immune response plays a critical role in defending against external or foreign antigens while also preserving tolerance to self-antigens. This equilibrium, referred to as immune homeostasis, is paramount for overall health. The regulatory mechanisms governing the maintenance of this delicate immune balance are notably complex. It is currently accepted that immune tolerance is a dynamic outcome regulated by multiple factors, including central and peripheral mechanisms. Its induction or elimination plays a significant role in autoimmune diseases, organ transplantation, and cancer therapy, markedly impacting various major diseases in modern clinical practice. Overall, our current understanding of immune tolerance is still very limited. In this review article, we summarized the main mechanisms that have been known to mediate immune tolerance so far, including endogenous immune tolerance, adaptive immune tolerance, other immune tolerance mechanisms, and the homeostasis of immune tolerance, identified the key factors that regulate immune tolerance, and provided new clues for immune system recovery in many autoimmune diseases, organ transplantation, and tumor therapy.

## 1. Introduction

The immune system of an organism faces the challenging task of maintaining protection against pathogens while also preserving tolerance to its own tissues [1]. Immune tolerance is a state in which the immune response to specific antigens, both self and nonself (foreign), is suppressed, and it is crucial for maintaining homeostasis and health within the body [2]. The specific function of immune tolerance varies depending on the conditions. For instance, under physiological conditions, individuals with a nonatopic constitution can maintain an immune tolerance toward allergens, thereby preventing allergic reactions and unnecessary immune responses to harmless substances. In pathological states, however, the immune system may develop tolerance toward pathogens, allogeneic transplants, or tumor antigens, leading to unwanted consequences as it may permit the progression of diseases [3].

In 1953, Billingham et al. [4] discovered that exposing the developing immune system to foreign antigens could induce specific immune tolerance, providing a theoretical basis for defining tolerance mechanisms and developing clinical tolerance induction methods. Subsequent research found that central tolerance mechanisms in adaptive immune responses eliminate potentially autoreactive cells through negative selection and preferential selection of high-affinity thymocytes by regulatory T cells (Treg cells) [3]; peripheral tolerance eliminates autoreactive cells that escape central mechanisms through clonal deletion and anergy. In summary, clonal deletion, clonal anergy, clonal ignorance, and regulatory immune cells collectively maintain the stability and tolerance of the immune system. The disruption of immune tolerance poses the organism at risk of severe autoimmune responses. Inducing allogeneic antigen-specific tolerance is crucial for preventing rejection in organ transplantation, highlighting the need for effective immunotherapeutic strategies. Research over the past decades has shown that neuropeptides and hormones produced by immune cells can be used to treat inflammation, autoimmune diseases, and transplant rejection [1, 5, 6]. Traditional suppression therapies like corticosteroids, methotrexate, and calcineurin inhibitors, though significantly improving the lives of organ transplant recipients and autoimmune disease patients, require lifelong treatment and nonspecifically suppress the entire immune system, posing considerable infection and cancer risks to patients [7]. The Immune Tolerance Network (ITN), founded in 1999 with sponsorship from the National Institutes of Health, primarily conducts research and evaluation of new tolerance-inducing therapies. Fourteen years ago, ITN had already carried out about 36 clinical trials and tolerance studies to explore innovative immune tolerance induction methods [7]. As of now, this number has increased to 75, which indicates that the field has garnered considerable attention and interest within the scientific community. However, establishing immune balance in organ transplantation, autoimmune diseases, and cancer treatment remains highly challenging, highlighting the complexities of inducing a long-term state of immune tolerance.

This review will primarily focus on self-tolerance, examining how the immune system recognizes and tolerates its own tissues to prevent the occurrence of autoimmune responses and summarizing a range of fundamental and newly discovered mechanisms of immune tolerance along with their clinical relevance based on the aforementioned research progress and background.

## 2. History of the Immune Tolerance Theory

**Figure 1:**
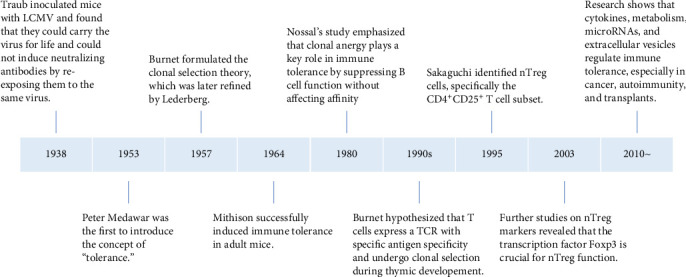
Immune tolerance theory and development timeline. LCMV, lymphocytic choriomeningitis virus; TCR, T cell receptor.

In 1938, Traub inoculated lymphocytic choriomeningitis virus (LCMV) into embryonic mice and discovered that these mice could carry the virus for life and could not induce neutralizing antibodies when attacked by the same virus later. In 1945, Owen observed that dizygotic twins with different genetic backgrounds possessed different blood type antigens. However, when the vascular systems of the twins' placentas were connected and their blood mingled, they did not reject each other but exhibited a natural union and symbiosis. Postbirth, each twin contained blood cells of a different blood type (chimerism) from the other twin. Billingham et al. [8] further found that if individuals are exposed to their specific antigens during the embryonic period, subsequent skin grafting between them does not provoke rejection, as immune tolerance had been induced during embryogenesis. Burnet et al. [9] postulated that the formation of this immune tolerance is related to the loss or inactivation of reactive lymphocytes caused by exposure to antigens during the early development of the immune system. Hence, in 1957, the “clonal selection” theory was proposed: different antigen recognition receptors of immune cells pre-exist in the body, and various antigens selectively activate corresponding receptors, triggering clonal activation, proliferation, and differentiation and generating a specific immune response. If the immune system is stimulated by antigens during the embryonic period, it can lead to clonal deletion or suppression, causing immune tolerance. In 1959, Lederberg et al. [10] refined the clonal selection theory, suggesting that immune cells carrying antigens in the body are crucial for generating immune tolerance or immune responses, depending on the maturity of the immune cells. Mitchison's [11] landmark study in 1964 demonstrated the induction of immune tolerance in adult mice through controlled antigen exposure, significantly advancing the understanding of immune regulation. In 1980, Nossal and Pike [12] administered fluorescein conjugated with human gamma globulin to neonatal or pregnant mice and examined the interaction between B cells and the tolerogen in the spleen 1–6 weeks after birth. Their findings revealed that, despite the functional suppression of B cells induced by both high and low doses of tolerogen, the affinity of these B cells remained unaffected. Based on this experimental observation, Nossal emphasized the significance of the concept of clonal anergy. In 1995, Sakaguchi et al. [13] first described CD4^+^CD25^+^ T cells, also known as Treg cells, which play a crucial role in maintaining peripheral immune tolerance and preventing excessive immune responses. In 2003, further research by Hori et al. [14] demonstrated that the Foxp3 gene encodes a transcription factor that is genetically defective in autoimmune and inflammatory syndromes in both humans and mice and is specifically expressed in naturally occurring CD4^+^ Treg cells. Moreover, Hori successfully transferred the Foxp3 gene into naive T cells via retroviral transduction, converting them into a phenotype similar to naturally occurring CD4^+^ Treg cells. Increasing attention is now being paid to how immune tolerance is regulated by various factors in the immune microenvironment, such as cytokines, metabolic states, microRNAs (miRNAs), and extracellular vesicles, particularly in the contexts of cancer, autoimmune diseases, and transplant tolerance [15, 16] (Figure 1).

## 3. Immune Tolerance Mechanisms

Immune tolerance is a dynamic process governed by a complex interplay of cytokines and genetic targets, which collectively coordinate the response of the immune system. Regulatory immune cells, particularly CD4^+^CD25^+^Foxp3^+^ Treg cells, are pivotal in maintaining and stabilizing immune tolerance [17]. These cells play a critical role in modulating the immune system's response to self and foreign antigens, thereby averting autoimmune diseases and promoting tolerance to allografts and self-antigens.

### 3.1. Endogenous Immune Tolerance

#### 3.1.1. The Role of Phagocytes in Self-Tolerance

Monocytes, the body's first line of defense against pathogens, can differentiate into macrophages with microbial phagocytic capabilities upon stimulation and secrete factors that regulate immune responses. The role of antigen-presenting cells (APCs) in maintaining immune tolerance is discussed below.

As a type of APC, macrophages play a crucial role in combating pathogens, maintaining normal tissue equilibrium, and self-antigen tolerance [18]. The activity of monocytes/macrophages is thought to be primarily regulated by miRNAs, which are involved in modulating the acquisition of immune tolerance in these cells [18]. miRNAs exert their regulatory effects through multifaceted mechanisms: for instance, miR-148a-3p can promote the differentiation of monocytes into macrophages and enhance the activation of M1-type macrophages via the Notch signaling pathway, upregulating sensitivity to bacterial lipopolysaccharide (LPS). Prolonged exposure of macrophages to LPS can lead these cells to acquire immune tolerance [19]. Furthermore, miRNAs can target intracellular signaling pathways, thereby affecting the function of monocytes/macrophages and consequently influencing the innate immune response levels of macrophages [20].

The clearance of apoptotic cells is crucial for maintaining immune homeostasis. If apoptotic cells are not promptly removed, they may release damage-associated molecular patterns (DAMPs), which can activate adjacent immune cells, leading to the disruption of immune homeostasis and the initiation of inflammatory responses [21]. Phagocytic cells can maintain self-tolerance by clearing apoptotic cells; upon engulfing apoptotic cells, phagocytes can secrete transforming growth factor-beta (TGF-β). TGF-β induces the generation of CD4^+^Foxp3^+^ Treg cells by binding to its receptors and activating the Smad signaling pathway, thereby promoting the formation of immune tolerance [22, 23]. Apoptotic cells can suppress the immune response to themselves in marginal zone macrophages (MZMs) by inducing the expression of indoleamine 2,3-dioxygenase (IDO), which inhibits T cell activation [24]. This indicates the bidirectional role of apoptotic cells in immune tolerance. Apoptotic cells in circulation are typically captured by phagocytes and transported to the marginal zone (MZ) of the spleen [25], where MZMs play a critical role in clearing apoptotic cells and maintaining tolerance [24, 26]. Mice lacking MZM exhibit immune responses to apoptotic cells, leading to autoimmunity [27]. The exact role of IDO expressed in the spleen, whether it reduces the presentation of self-antigens to potentially autoreactive T cells or actively suppresses these T cells after antigen presentation, remains unclear [24]. Another potential mechanism for inducing tolerance in MZM involves the signaling pathway between the liver X receptor (LXR) and IDO [26]. Mice lacking LXR can develop autoantibodies and autoimmune glomerulonephritis, while LXR agonists can improve disease progression in lupus-like autoimmune mouse models [24]. LXR regulates the expression of genes such as MARCO, SIGN-R1, TIM4, and CD169, which are vital for the transcriptional response of macrophages during the engulfment of apoptotic cells and can regulate the intracellular homeostasis of MZM [28, 29]. Additionally, marginal zone metallophilic macrophages (MMM) express the SER-4 molecule, which recognizes sialoadhesin (CD169), and the CD169-expressing cell subpopulation has a unique function in engulfing apoptotic cells and inducing immune tolerance [24]. Therefore, it is speculated that LXR signal can promote the self-renewal of MZM and the maintenance of immune tolerance during the signaling process of apoptotic cells [26]. Red pulp macrophages (RPMs) and tingible body macrophages (TBMs) within the MZ are primarily responsible for engulfing and clearing apoptotic B cells during maturation [30, 31]. TBMs facilitate the rapid clearance of apoptotic cells by inducing and binding to molecules on the cell surface via milk fat globule epidermal growth factor 8 (MFG-E8) [32]. Mice deficient in MFG-E8 exhibit an inability of TBM to recognize and clear apoptotic lymphocytes, leading to spontaneous production of autoantibodies and systemic autoimmune disorders [33]. These studies highlight the critical role of rapid clearance of apoptotic cells in maintaining self-immune tolerance. Furthermore, the nuclear factor kappa B (NF-κB) pathway is considered important in the activation of dendritic cells (DCs) by microbial stimuli and proinflammatory cytokines, with IKKβ being a key regulatory factor for NF-κB activation. Deficiency of IKKβ in DCs can lead to uncontrolled immune homeostasis and spontaneous autoimmunity, underscoring the significance of NF-κB in immune tolerance and homeostasis [34].

#### 3.1.2. The Role of Natural Killer (NK) Cells in Self-Tolerance

NK cells, lymphocytes developed from bone marrow precursors, are the body's first line of defense, capable of killing virus-infected or malignant host cells without prior sensitization [35]. Highly heterogeneous, NK cells play significant roles in promoting transplant rejection and antitumor responses [36]. Unlike T and B cells, NK cells do not express antigen receptors but exhibit immunological characteristics against various cells through a range of activating and inhibitory receptors [37, 38]. Harmon et al. [36] emphasized that NK cells play crucial and distinct roles in liver transplantation based on whether they are derived from the recipient or the donor. Compared to peripheral blood NK cells, liver-resident NK cells (recipient-derived) produce more granzyme and perforin and serve as an early source of interferon-gamma (IFN-γ), driving alloreactive T cell responses during graft rejection [36]. High levels of IFN-γ activate hepatic sinus epithelial cells to release chemokines that recruit T cells and other immune cells, leading to increased inflammation [39]. However, activated donor NK cells can directly lyse or kill recipient immune cells that initiate or mediate organ rejection, such as T cells [40, 41] and immature DCs [42], which allows them to partially contribute to immune tolerance. Similar to the liver, NK cells in the uterus possess a complex immune system and diverse functions, which are now considered fundamental for achieving embryo implantation and successful pregnancy [43]. Uterine NK (uNK) cells are classified into endometrial NK (eNK) cells and decidua NK (dNK) cells. Notably, dNK cells express multiple inhibitory NK cell receptors (iNKRs), including killer cell immunoglobulin-like receptors (KIRs), leukocyte immunoglobulin-like receptors, and C-type lectin-like receptor families (NKG2/CD94). These receptors specifically bind to nonclassical major histocompatibility complex class I (MHC I) molecules on the surface of trophoblast cells, thereby mediating recognition and immune tolerance functions in the embryo [44].

Normal cells can express ligands that activate NK cells, underscoring potential autoreactivity. However, normal cells avoid NK cell-mediated autoreactivity by engaging MHC I receptors with NK cell-expressed inhibitory receptors [45]. Most NK cells express at least one self MHC I-specific receptor to maintain self-tolerance [37, 46], but some NK cells show tolerance despite lacking any MHC I-specific inhibitory receptors [47], suggesting mechanisms of self-tolerance that are both dependent and independent of MHC I [46]. NK cells encountering MHC I-expressing cells receive signals that induce an unresponsive state, although it is unclear if this involves adjusting NK cell response thresholds [48].

NK cells that mature in an environment with MHC I molecules can express MHC I-specific inhibitory receptors. According to the report of Jaeger and Vivier [46], NK cells matured in an MHC I-rich environment can express specific inhibitory receptors, recognizing self MHC I molecules and blocking activation. This ensures that mature NK cells express at least one such receptor [37]. NK cells lacking these receptors might achieve immune tolerance by downregulating activating receptors or inhibiting signaling pathways. Activation and inhibition of NK cell receptors occur within actin-dependent reticular structures on the cell membrane. Inhibiting NK cell activation pathways raises their activation threshold, preventing autoreactivity and reducing target cell killing in immune homeostasis [46]. Moreover, NK cell hyporesponsiveness can be achieved by shutting down stimulating pathways, indicating potential regulatory mechanisms modulating NK cell reactivity [37].

### 3.2. Adaptive Immune Tolerance

#### 3.2.1. Central Tolerance

In the thymus, central tolerance is established by eliminating most self-reactive cells, effectively reducing the tendency for autoreactivity in mature peripheral immune cells [49]. This process is crucial for preventing autoimmune responses, as it ensures that T cells recognizing self-antigens are selectively deleted during their development in the thymus, thereby maintaining immune system balance and self-tolerance [50].

##### 3.2.1.1. T Cell Central Tolerance

Pre-T cells in the bone marrow travel through the bloodstream to the thymus, where they further differentiate into mature T cells. The T cell receptor (TCR) on the surface of T cells interacts with autologous antigen peptides–MHC ligands on thymic cortical epithelial cells (cTECs). T cells with high affinity for these ligands receive survival signals from cTECs, a process known as positive selection, which allows them to survive; otherwise, apoptosis occurs. This selection process endows mature CD8^+^CD4^−^ T cells with the ability to recognize antigen peptide fragments and their own MHC I/II molecular complexes, forming the basis for the MHC restriction phenomenon of T cells. After positive selection, T cells undergo further screening based on the affinity of their TCRs for autologous peptides. At the corticomedullary junction, DCs and macrophages express high levels of MHC I/II antigens. Autoantigen components can form complexes with MHC I/II antigens on the surface of DCs or macrophages [51]. If, after positive selection, thymocytes recognize the autoantigen–MHC complexes on the surface of DCs or macrophages, they can develop self-tolerance and halt further development. The thymocytes that cannot recognize the complex of autoantigen and MHC can continue to develop into CD4^+^CD8^−^ or CD4^−^CD8^+^ single positive cells, which leave the thymus and migrate to the peripheral blood [30]. Effective negative selection depends on the presence of autogenic peptides in the medulla of the thymus, because antigens must be present in the thymus to induce central tolerance [52], and the source of autoantigens in the thymus has been one of the focal points of debate. First, different APCs in the thymus express different self-proteins, and thymus medullary epithelial cells (mTECs) express tissue-limiting antigens under the control of autoimmune regulator (Aire) transcription factors [51]. Second, blood-borne myeloid DCs (MDCs) and plasmacytoid DCs (pDCs) can introduce peripheral antigens into the thymus [53]. Third, the antigen processing mechanism of MHC I and MHC II is different among different APCs in the thymus, which affects the production of antigen epitopes [54]. In the past decade, the role of Aire in negative selection has been discovered. Aire is selectively expressed primarily by mTECs and induces negative selection of T cells by modulating the expression of tissue-specific antigen (TSA) in the thymus [3]. In addition, thymus DC is also involved in T cell negative selection. The study of Thomas Brocker et al. [55] showed that DC expressing specific MHC II molecules can eliminate CD4^+^ T cells that react with their own antigens, thus inducing negative selection of CD4^+^ T cells. In human, thymic stromal lymphopoietin (TSLP), which can stimulate the maturation of thymus DC, is mainly responsible for inducing the proliferation and differentiation of Foxp3^+^ Treg cells [3]. Similarly, DC-deficient mice are unable to perform normal negative selection, resulting in a large number of self-reactive CD4^+^ T cells, leading to autoimmune diseases [56].

##### 3.2.1.2. B Cell Immune Tolerance

In the bone marrow, B cells establish their initial repertoire of antigen receptors during early development through the process of V(D)J recombination of immunoglobulin (Ig) germline genes, resulting in a diverse array of B cell receptors (BCRs). Each BCR is capable of specifically recognizing distinct antigens. The formation of this repertoire is a pivotal aspect of B cell maturation and forms the foundation of adaptive immune responses [57]. During B cell maturation, a selection process ensues, comprising both positive and negative selection, to ensure that B cells do not react against self-antigens, thereby constituting a component of immune tolerance. Positive selection retains B cells that can recognize self-antigens presented by self-major MHC molecules, while negative selection eliminates B cells with high affinity for self-antigens. This ensures that the mature B cell repertoire consists of cells capable of recognizing nonself antigens and mounting an appropriate immune response while simultaneously avoiding attacks on self-tissues [58]. Immature B cells in the bone marrow microenvironment predominantly encounter self-antigens through their BCRs. The specificity or affinity of BCRs for self-antigens determines the deletion or inactivation of autoreactive B cells [59, 60]. Receptor editing is a primary mechanism for removing autoreactive B cells from the repertoire, where binding of BCRs to self-antigens allows ongoing immunoglobulin gene recombination to alter the specificity of B cells carrying self-reactive antigen receptors [61]. Secondary rearrangement of the light chain gene in autoreactive IgM-positive immature B cells is another critical regulatory mechanism. This process allows immature B cells that bind to self-antigens to undergo secondary rearrangement at the light chain locus, generating new specificities with lower affinity for self-antigens. Under normal conditions, B cells with high affinity for self-antigens undergo central tolerance mechanisms in the bone marrow and are eliminated through negative selection. However, under special conditions, they may migrate to the spleen, where their BCRs engage with self-antigens of varying affinities [62]. For instance, some B cells may not encounter sufficient self-antigens in the bone marrow, or their BCRs may not have a high enough affinity for self-antigens to trigger tolerance mechanisms, thereby allowing these B cells to evade tolerance and migrate to the spleen to bind with self-antigens of diverse affinities. Certain special B cell subsets, such as B-1 cells, can bypass this selection and escape to the spleen to exert distinct tolerance mechanisms [63]. Furthermore, in certain autoimmune disease states, this selection mechanism may be disrupted, increasing the risk of developing B cell-mediated autoimmune diseases [64]. Strong binding can lead to follicular exclusion of B cells, while intermediate binding can result in enhanced internalization of IgM and loss of function [65]. Research by Zikherman, Parameswaran, and Weiss [66] found that in mice, B cells can rapidly induce the expression of a green fluorescent protein (GFP) transgene under the transcriptional control of the nuclear receptor 77 (nur77) regulatory element upon antigen–BCR engagement. Surprisingly, all mature B cells expressed the nur77-driven GFP, albeit at varying intensities, indicating that all B cells are autoreactive to some extent. This suggests that each individual possesses a vast repertoire of autoreactive B cells, potentially capable of differentiating into plasma cells and secreting highly autoreactive pathogenic autoantibodies [67, 68].

#### 3.2.2. Peripheral Tolerance

Peripheral tolerance serves as an additional safeguard to eliminate or suppress autoreactive cells that escape central tolerance, achieved through further clonal deletion or active suppression by Treg cells. While clonal deletion is effective in eliminating most autoreactive cells, the presence of peripheral autoreactive cells indicates that some self-reactive cells can evade this process [69]. This necessitates the existence of other mechanisms to ensure peripheral tolerance, ensuring that the immune system does not attack the body's own tissues [70, 71]. These mechanisms include the induction of anergy in T cells, where they become inactive in the absence of proper costimulatory signals and the suppression of autoreactive cells by Treg cells, which play a crucial role in maintaining immune homeostasis and preventing autoimmune responses.

##### 3.2.2.1. The Molecular Mechanisms of Immune Tolerance: Clonal Anergy and B Cell Exclusion

In 1980, Nossal and Pike [12] first introduced the concept of clonal anergy, where immune cells are inactivated and unable to mount an immune response against corresponding self-antigens, a state known as immunological anergy. This phenomenon can occur in both T and B cells within peripheral immune organs. T cell tolerance begins with the formation of the TCR on the membrane of thymic T cell precursors [72]. T cell anergy, distinct from apoptosis induction, is characterized by reduced cell proliferation and cytokine production upon restimulation [73], often accompanied by impaired proliferation and IL-2 secretion. Anergy in T cells is thought to be induced by antigen presentation in conjunction with CD28 costimulation or high coinhibition, characterized by low IL-2 production and cell cycle arrest at the G1/S phase [74]. Furthermore, chronic infection promotes breaking of clonal anergy of T cells by activating the expression of CD80/CD86 on APCs presenting self-antigen complexed with MHC. Early growth response gene 2 (Egr2) may act as a central transcription factor regulating the anergic state in T cells [75]. The single binding of MHC to TCR, promoting Ca2^+^ imbalance and cytoplasmic retention of active RAP-1, is corrected by CD28 costimulation. Incorrect signaling downstream of the TCR/CD28 pathway, particularly in mTOR and RAS/MAPK pathways, contributes to the induction of anergy [76, 77].

For B cells, anergy is characterized by reduced responsiveness to BCR and TLR signaling [78]. Various factors influence the timing and mechanisms of B cell anergy, such as the nature and immunogenicity of self-antigens, BCR affinity for the antigen, and the timing of antigen encounter during B cell differentiation [79]. Anergy in B cells is primarily attributed to reduced surface IgM-type BCR expression. Schmidt and Cyster [80] found that anergic B cells cannot enter follicles, remaining at the T–B cell interface, a phenomenon known as follicular exclusion. Anergic B cells, due to follicular exclusion, lack interactions with T cells, maintaining an immunotolerant state [81]. Continuous binding of self-antigen to BCR is crucial for sustaining B cell anergy, promoting monophosphorylation of ITAM tyrosine residues, limiting SYK aggregation and activation [82]. The activation of inhibitory complexes like SHIP1-DOK1, triggered by ITAM monophosphorylation and LYN activation, reduces intracellular PtdIns(3,4,5)P3 levels [81]. Upregulation of the PTEN gene in anergic B cells also lowers PtdIns(3,4,5)P3 levels, decreasing extracellular calcium influx and inhibiting activation pathways such as NFAT, NF-κB, JNK, and CARD11, thus maintaining the immunotolerant state of anergic B cells [83].

##### 3.2.2.2. Molecular Mechanism of Immune Tolerance and Clonal Ignorance

Immune ignorance refers to the coexistence of T and B cells with self-antigens without triggering an immune response. In animal studies, similar tolerance models exhibit different mechanisms of tolerance. Research by Kurts et al. [84] demonstrate that the level of antigen expression plays a crucial role in determining the outcome of tolerance. When ovalbumin (OVA) is expressed at low levels, mice exhibit immune ignorance; at high expression levels, OVA-specific CD8^+^ T cells are selectively deleted [84].

**Figure 2:**
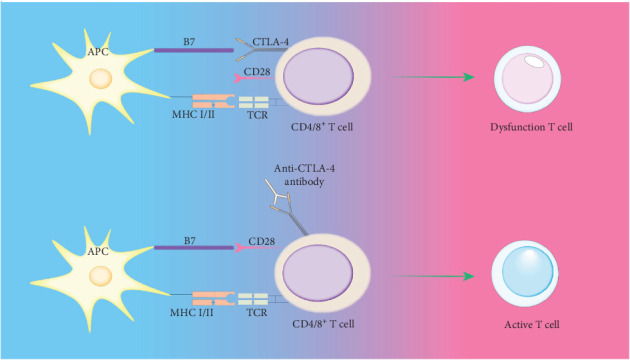
T cell stimulation signal loss and inhibitory molecular binding diagram. APC, antigen-presenting cell; CTLA-4, cytotoxic T-lymphocyte antigen 4; MHC I/II, major histocompatibility complex class I/II; TCR, T cell receptor.

**Figure 3:**
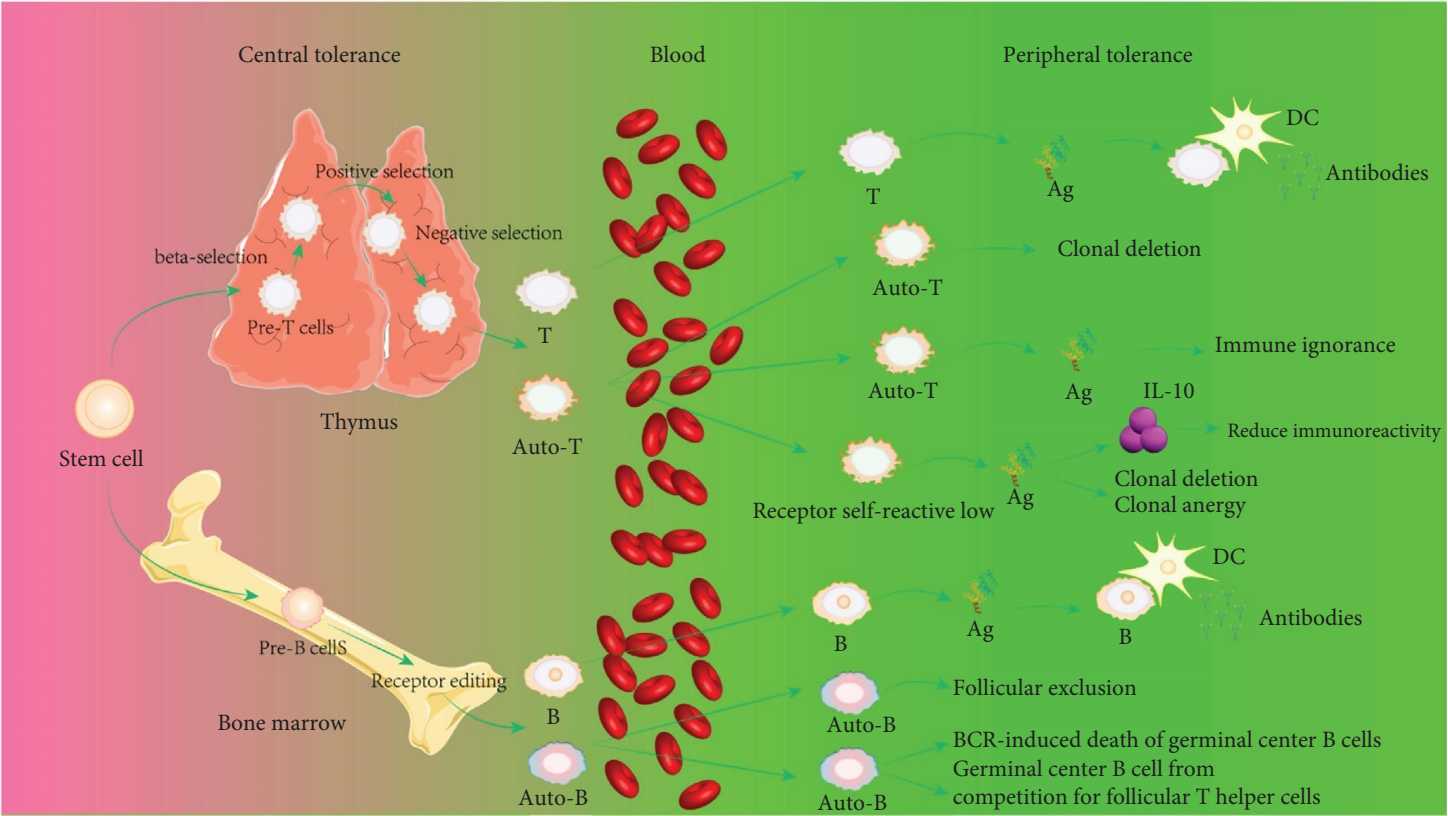
Map of central and peripheral immune tolerance mechanisms. Ag, antigen; Auto-B, autoreactive B cells; Auto-T, autoreactive T cells; B, B cells; DC, dendritic cells; T, T cells.

Activation of immune cells requires various stimulatory signals, and the absence of these signals can lead to immunological anergy or ignorance. For instance, if the concentration of self-antigen is too low or its immunogenicity is weak, failing to provide a strong primary activation signal, it can result in immune ignorance or anergy. When the expression of costimulatory molecules (such as B7, LFA-1, and CD40) by APCs is abnormal, leading to insufficient provision of the second signal, immunological anergy can occur. The inability of self-antigens to be effectively processed and presented by self-APCs can also lead to immune ignorance. The mechanisms of immune tolerance can vary due to the absence or abnormality of immune signals. Additionally, the mechanism of immunological anergy includes the binding of the inhibitory molecule cytotoxic T-lymphocyte antigen 4 (CTLA-4), expressed on T cells, with B7 molecules, releasing inhibitory signals that render T cells unresponsive [72] (Figure 2). Immune-privileged organs are specific body regions, such as the brain and the anterior chamber of the eye, where transplanted tissues typically do not trigger immune rejection. Initially, it was believed that immune-privileged organs prevent immune responses by restricting antigen leakage. However, research has shown that certain antigens, previously thought to be sequestered, can interact with T cells upon exposure to the immune system, without eliciting a destructive immune response, instead inducing immune tolerance or nondamaging responses [85]. For example, the zonadhesin (ZAN) antigen in the sperm acrosome usually acts as a sequestered antigen, but when exposed by vasectomy, it can rapidly induce testis antigen-specific immune tolerance. However, if there is partial depletion of Treg cells, this tolerance is disrupted, leading to bilateral experimental autoimmune orchitis (EAO) and the production of antibodies against ZAN [86] (Figure 3).

Picture annotation: T cell activation requires dual signaling stimulation. APCs directly interact with T cells, presenting antigens to the TCR complex via MHC I/II molecules, which generates the first signal for T cell activation. The adhesion molecules on APCs bind to those on CD4^+^ T cells, providing the second signal necessary for T cell activation. CTLA-4 interacts with B7 molecules, releasing inhibitory signals that can render T cells anergic, placing them in a state of nonresponsiveness.

##### 3.2.2.3. Molecular Mechanism of Immune Tolerance Between Foxp3^+^ Treg Cells and TGF-*β*

In 1995, Sakaguchi et al. first introduced the concept of Treg cells, suggesting that immune responses could be controlled by a specialized subset of suppressive T cells. Treg cells are a group of cells that regulate potentially harmful self-reactive T cells. Treg cells are categorized into natural Treg cells (nTreg cells), which originate from the thymus and primarily exert their suppressive function through cell contact mechanisms, and induced Treg cells (iTreg cells), which are derived from CD4^+^ T cells in the presence of certain cytokines like IL-10 or TGF-β, or specific drugs, upon antigen exposure. The specific expression of the transcription factor Foxp3 and intracellular markers defines the phenotype and function of Treg cells [87].

**Figure 4:**
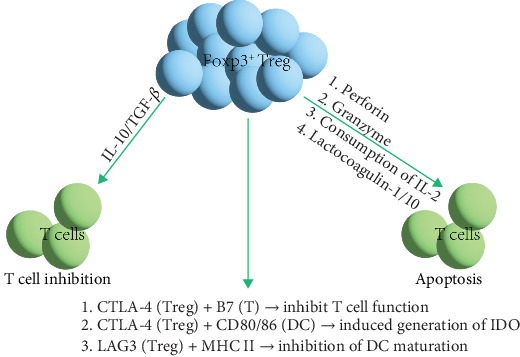
The mechanism of Foxp3^+^ Treg cells inducing immune tolerance. CTLA-4, cytotoxic T-lymphocyte antigen 4; DC, dendritic cells; IDO, indoleamine 2,3-dioxygenase; MHC I, major histocompatibility complex class I; Treg, regulatory T cells.

Foxp3^+^ Treg cells regulate immune responses and induce immune tolerance through three main mechanisms, as shown in Figure 4: (1) inducing apoptosis in effector T cells (Teff) [88]; (2) suppressing the activity of Teff; and (3) acting on APCs to inhibit their function. Treg cells inhibit immune responses in four ways, including the secretion of inhibitory cytokines like TGF-β, which is expressed on the surface of activated Treg cells and exerts immunosuppressive effects through cell membrane contact [89]; IL-10 suppresses immune activity by inhibiting APC functions. Treg cells can also kill B cells in a granzyme B-dependent manner and partially in a perforin-dependent manner, inhibiting B cell activity [88]. Moreover, Treg cells can exert suppressive effects through metabolic disruption and regulating the maturation or function of DCs. Peripheral T cell tolerance is primarily regulated by the cytokine TGF-β and Foxp3^+^ Treg cells. However, whether TGF-β and Treg cells belong to the same regulatory pathway or operate through different mechanisms remains unclear [90]. TGF-β promotes the development of thymic Treg cells (tTreg cells) and the survival of tTreg precursors, inhibits T cell clonal deletion and peripheral Treg cell differentiation, and induces Foxp3 expression [91, 92]. T cell tolerance induction by TGF-β primarily occurs through a Foxp3-independent mechanism, which plays a leading role in regulating peripheral T cell tolerance [90]. Deficiency in TGF-β cytokines can lead to a lack of thymic tTreg differentiation [93]. Thus, TGF-β is crucial for maintaining immune tolerance and regulating self-reactive T cells. Although Treg cells can secrete TGF-β, TGF-β independently suppresses self-reactive T cells separate from Foxp3^+^ Treg cells. Both TGF-β and Foxp3^+^ Treg cells play significant roles in maintaining peripheral tolerance. In the context of transplant tolerance, research has found that macrophages expressing DC-SIGN, a type II transmembrane C-type lectin with a carbohydrate recognition domain, expressed in human DCs and macrophages and involved in various aspects of immune responses, are essential for inducing suppressive tolerance [94]. Monocytes differentiated in vitro in the presence of M-CSF and IL-4 to induce DC-SIGN expression act as inefficient inducers of allogeneic mixed lymphocyte reactions [94]. Macrophages expressing DC-SIGN stimulated with M-CSF and IL-4 in vitro can drive Treg expansion and induce tolerance in allogeneic naive CD4^+^ T cell precursors, whereas other macrophages treated with GM-CSF and IL-4 cannot drive Treg expansion [94]. Treg cells employ a set of distinct mechanisms to enhance peripheral tolerance, reflecting the complexity and plasticity of immune responses.

In addition, avoiding the targeting of Treg cells by Teff is also critical for maintaining immune tolerance. Treg cells employ a variety of strategies to evade targeting by Teff. They can secrete inhibitory cytokines such as IL-10 and TGF-β, which aid in suppressing the activation and proliferation of Teff, thus maintaining immune tolerance and preventing autoimmune diseases [95]. IL-2 is a pleiotropic cytokine in immune regulation, exerting opposite effects on Treg cells and Teff; therefore, Treg cells competitively consume IL-2 to inhibit the growth and activity of Teff [96]. Treg cells express CD39 and CD73 to convert ATP into adenosine, which exerts immunosuppressive effects through the adenosine A2A receptor pathway, even in an apoptotic state, effectively avoiding targeting by Teff. Treg cells share phenotypic characteristics with cytotoxic lymphocytes, allowing them to avoid being targeted and suppressed without affecting effective antitumor immunity in tumor environments [97, 98]. These mechanisms work in concert to enable Treg cells to perform their immunosuppressive function in the immune system while avoiding targeting by Teff.

Foxp3^+^ Treg cells induce immune tolerance through three mechanisms: Firstly, Foxp3^+^ Treg cells can directly kill Teff through perforin or granzymes. They also express CD25 and the high-affinity IL-2 receptor on their surface, inducing T cell apoptosis by consuming IL-2 and competing with Teff for IL-2 [99]. Secondly, Foxp3^+^ Treg cells transfer cAMP to T cells through gap junctions, reducing IL-2 levels. Additionally, they produce soluble immunosuppressive molecules, such as IL-10 and TGF-β, to inhibit T cell functions [99]. Lastly, Foxp3^+^ Treg cells express CTLA-4, which competes with CD28 on T cells for binding to B7 molecules on APCs, inhibiting the costimulatory signals required for T cell activation and proliferation. The interaction of CTLA-4 with CD80/CD86 on DCs induces the production of IDO, weakening T cells' ability to attack host tissues. Foxp3^+^ Treg cells also express lymphocyte-activation gene-3 (LAG3), which binds to MHC II molecules, inhibiting DC maturation and exerting an immunosuppressive effect [99].

##### 3.2.2.4. Activation-Induced Cell Death (AICD) Molecular Mechanism

Peripheral autoreactive cell apoptosis is primarily induced through AICD. Following negative selection of autoreactive T cells, AICD can eliminate overly activated autoreactive cells after an immune response, inducing peripheral tolerance. The main mechanism of AICD is the cascade effect of Fas signaling, where cells expressing Fas (CD95/APO-1) interacting with cells expressing Fas ligand (FasL), such as activated T cells [100], can activate intracellular caspase8, triggering the activation of downstream caspase3, thus initiating a series of cascade reactions that activate the nuclear apoptosis program. The activation of caspases, chromatin condensation, and DNA fragmentation are key features of AICD [101]. FasL-mediated apoptosis is caspase-dependent and considered crucial for maintaining tolerance and stability in T and B cells. The functionality of Fas and FasL is essential for AICD in mature T cells ex vivo [102]; mice or humans with a deficiency or mutation in Fas or FasL can develop severe autoimmune diseases, such as lupus-like syndrome. Researchers like Schlomchil found that in mice lacking functional Fas or FasL, B cells are key drivers of autoimmunity; ablating B cells or selectively restoring Fas expression in them can prevent autoimmunity in mice [103]. In T cell-dependent B cell activation, Teffs expressing CD40L can bind to CD40 on activated B cells, inducing the upregulation of Fas expression [1]. Besides Fas-mediated AICD, T cells can also induce p73-dependent AICD in mature T cells through the activation of E2F-1 [104]. The relationship between Fas and FasL-mediated AICD and p73-dependent AICD remains unclear [105]. The expression of the super-repressor of NF-κB activation inhibitor IkBaM also triggers apoptosis during T cell activation [106]. Moreover, Fas-mediated anergy is not a universal mechanism for immune tolerance, as not all anergic B cells express Fas.

##### 3.2.2.5. Mucosal Immune Tolerance

The mucosal immune system employs two anti-inflammatory strategies: immune exclusion, which controls microbial colonization on the epithelium and inhibits the infiltration of potentially harmful substances through the secretion of antibodies, and immune suppression, which offsets local and peripheral hypersensitivity reactions to harmless antigens (like food proteins), known as oral tolerance [107]. Oral and nasal tolerance can suppress various autoimmune diseases in animal models, such as experimental allergic encephalitis (EAE), uveitis, thyroiditis, and nonautoimmune diseases like asthma, atherosclerosis, colitis, and stroke [108]. Mucosal tolerance involves multiple mechanisms, with antigen dosage being a crucial determinant [109]. High antigen doses favor clonal anergy/deletion, while low doses promote active suppression, with low-dose antigen preferentially inducing Treg cells to secrete downregulatory cytokines like TGF-β, IL-10, and IL-4 [109]. Th3-type Treg cells are dependent on TGF-β, and TR1-type Treg cells rely on IL-10 and are induced by nasal antigens, while Foxp3^+^ iTreg cells are induced by oral antigens and aryl hydrocarbon receptor ligands [110]. In TCR transgenic mice, orally delivered antigen also leads to a relative increase in CD4^+^CD25^+^ Treg cells expressing CTLA-4 or Foxp3 [108]. Studies have shown the role of intestinal DCs in oral tolerance, with oral antigens primarily recognized by DCs in mesenteric lymph nodes (MLNs). Mucosal DCs can induce Foxp3^+^ Treg cells by producing TGF-β. Research by Worbs et al. [111] indicates that CCR7-deficient mice cannot induce oral tolerance, as CCR7 deficiency prevents DC migration from the intestine to MLNs, showing that immune-related antigens are transported in a cell-associated manner. In oral tolerance, the expression of CD11b in intestinal DCs increases, producing IL-10 and IL-27 to enhance IL-10 production by Treg cells [108]. Costimulatory molecule pathway studies have found that CD80 is essential for inducing low-dose oral tolerance. In a peanut allergy model, animals treated with CTLA-4Ig, anti-CD86, or anti-CD80 plus anti-CD86 achieved successful oral tolerance, but those treated with anti-CD80 were compromised [112]. This underlying mechanism may be associated with the regulation of T cell activation and tolerance, particularly Treg cells, which may contribute to alleviating allergic reactions and promoting immune tolerance by affecting the number and function of Treg cells as well as the secretion of anti-inflammatory cytokines, but further research is needed to clarify.

### 3.3. Homeostasis of Immune Tolerance

The primary mechanism underlying the development and progression of autoimmune diseases is the immune system's failure to regulate immune tolerance mechanisms [24]. Both central and peripheral tolerance pathways are influenced by a combination of factors, including polymorphisms in autoimmune susceptibility genes, regulatory cell subpopulations, intrinsic T cell regulatory mechanisms, pathogenic cell subpopulations, and environmental and immunological factors that depend on the site of the immune response and the individual's immune status. The balance between inflammatory and regulatory signals ultimately determines the outcome of autoimmunity and tolerance [3]. The table summarizes key factors in immune tolerance. Moreover, increasing evidence shows that tolerant T cells exhibit unique transcriptional characteristics. Many transcription factors associated with tolerance have been identified, including Egr-2, Egr-3, Ikaros, CREM, p50, ZEB1, Blimp-1, Tob, and Smads, all of which can be considered biomarkers of the tolerant state [8]. The multilevel plasticity of immune tolerance is crucial for establishing tolerance. Various factors can impact the establishment of immune tolerance, including the route and location of antigen presentation, the source and form of the antigen (dose, with or without adjuvants/inflammation, tissue antigens, etc.), the cytokine environment, the interplay between intrinsic T cell signaling and extrinsic regulation, genetic background, the immune environment, and the plasticity of tolerance [3] (Table 1).

### 3.4. Other Immune Tolerance Mechanisms

#### 3.4.1. Ego–Non-Self-Identification Principle

The principle of self-nonself recognition by the immune system was established by Paul Ehrlich at the beginning of the last century. The concept of self-nonself discrimination might be an illusion because the immune system itself does not have inherent self-recognition encoded in its genome. Instead, it develops a repertoire of cells through clonal deletion processes, where “self” is defined as what the immune system does not respond to, and self and nonself can interconvert. Developing lymphocytes randomly express specific antigen receptors, and during a critical period, if the antigen receptor is engaged, the cell is deleted; otherwise, the cell matures [105].

#### 3.4.2. Idiotypic–Anti-Idiotypic Network Theory

In 1974, Danish immunologist Niels Jerne [117], building on the concept of clonal selection, proposed the renowned idiotype network theory. This theory posits that during an immune response, the variable region (V region) of an antibody molecule A1 induced by an antigen possesses a unique idiotype (Id) structure. This Id can specifically stimulate the body to produce an anti-idiotype (Anti-Id) antibody A2, whose Id in turn stimulates the production of another complementary Anti-Id antibody and so on. The theory is based on the mutual recognition of Id and Anti-Id, forming an Id–Anti-Id network within the immune system that recognizes, stimulates, and regulates each other, effectively modulating the immune response [117].

#### 3.4.3. Immune Recognition of Danger Theory

Immunologist Polly Matzinger [118] proposed the danger theory in 1994, which suggests that the immune system functions not by distinguishing “self” from “nonself,” but by recognizing “danger signals.” Matzinger posited that the signals initiating immune recognition are danger signals produced when the body's cells are damaged. Any factor causing harm to the body can initiate the immune recognition process, where danger signals promote the activation of resting APCs, leading to an immune response. In the absence of danger signals, T cells become tolerant to the antigen [119–121]. This theory contrasts with the self-nonself paradigm, which posits that foreignness alone can trigger an adaptive response [122]. In the danger model, the key determinant for initiating an immune response is the presence of antigens within tissue damage; without damage or if cells die by apoptosis without injury, no immune response is generated. Conversely, if cells are damaged, it can provoke an immune response [118].

## 4. Conclusions and Future Directions

Immune tolerance is not the result of a single cell type but rather a complex interplay among multiple cells and factors. While current models of central and peripheral tolerance explain much of the mechanisms for the formation and maintenance of immune tolerance, and there may be commonalities across different stages of immune tolerance, the complete picture of immune tolerance mechanisms is still not fully understood. With advances in clinical cancer treatment and organ transplantation in surgical clinics, significant progress has been made in studying the molecular mechanisms of immune tolerance, raising higher demands for understanding these mechanisms.

By summarizing the existing literature on immune tolerance mechanisms in this article, it can be observed that extracellular vesicles (such as exosomes) and miRNAs play a crucial role in immune tolerance. Extracellular vesicles serve as important mediators of intercellular communication, capable of carrying molecules such as proteins, lipids, and nucleic acids, including miRNAs. These molecules can modulate the biological functions of recipient cells and may play a significant role in regulating the behavior of immune cells and immune responses within the study of immune tolerance. Therefore, they represent an important direction for future research in the field of immune tolerance, offering new hope for the treatment of many autoimmune diseases, organ transplants, and cancers.

## Figures and Tables

**Table 1:** The role of various factors in immune tolerance factors related to induction of immune tolerance.

Key factors in immune tolerance	Role in immune tolerance
Regulatory cells	
Regulatory T cells	Secrete IL-10, TGF-β; express CTLA-4; consume IL-2
Regulatory B cell	Secrete IL-10, IL-35, TGF-β
Regulatory DCs	Secrete IDO; participate in clonal anergy when they do not express CD80/CD86
Tfh	Regulate germinal center and humoral immunity [113]
MDCs	As an inhibitory population of myeloid cells, MDCs, as an effective negative regulator of immune response [114], can block costimulation and induce immune tolerance after heart transplantation
Gene target	
PD-1	As a negative regulator to maintain peripheral tissue tolerance
CTLA-4	Encodes a receptor on the surface of T cells that is involved in controlling T cell proliferation and regulating apoptosis
HLA-II	It is expressed in APC and is closely related to autoimmune injury of islet B cells
Fox gene	Foxp3 is expressed in CD4^+^CD25^+^ Treg, and through inhibition of effector cells, abnormal Foxp3 expression is induced by autotolerance
Immunomodulator	
IL-2	A key factor in T cell expansion that intrinsically promotes T cell tolerance by supporting Fas-mediated apoptosis [3]
IL-10	Inhibit the activity of APC
TGF-β	Promote the development of thymus Treg and the survival of precursor tTreg, inhibit the deletion of T cell clone and peripheral Treg differentiation, and induce Foxp3 expression
Aire	Regulate the expression of central and peripheral TSA and influence negative selection
B7S1	Expressed in lymphoid and nonlymphoid tissues, it is a negative costimulatory factor that regulates the threshold of T cell activation [115]
MHC I/Ⅱ	As variants of HLA expressed in DCs can present self-antigens [116]
Neuropeptide	
VIP, α-msh	Promote the activation of naive CD4^+^CD25^−^ T cells or induce peripheral production of Foxp3^+^ Treg cells [1]
Urocortin, adrenomedullin, cortistatin	Inhibit Th1 response and induce Treg [1]

Abbreviations: Aire, autoimmune regulator; APC, antigen-presenting cell; CTLA-4, cytotoxic T-lymphocyte antigen 4; DCs, dendritic cells; IDO, indoleamine 2,3-dioxygenase; MDCs, myeloid dendritic cells; MHC I/II, major histocompatibility complex class I/II; TGF-β, transforming growth factor-beta; TSA, tissue-specific antigen.

## Data Availability

As this article is a review, no original datasets were generated or analyzed. All data referenced in this manuscript are publicly available through the cited sources. Readers can access the data via the corresponding references and online databases as indicated in the manuscript.
